# Effects of *NR1I2* and *ABCB1* Genetic Polymorphisms on Everolimus Pharmacokinetics in Japanese Renal Transplant Patients

**DOI:** 10.3390/ijms231911742

**Published:** 2022-10-03

**Authors:** Hironobu Yagishita, Hideaki Kagaya, Mitsuru Saito, Kazuyuki Numakura, Ryohei Yamamoto, Ryuichiro Sagehashi, Tomonori Habuchi, Shigeru Satoh, Masatomo Miura

**Affiliations:** 1 Department of Pharmacy, Akita University Hospital, 1-1-1 Hondo, Akita 010-8543, Japan; 2 Department of Urology, Akita University Graduate School of Medicine, 1-1-1 Hondo, Akita 010-8543, Japan; 3 Center for Kidney Disease and Transplantation, Akita University Hospital, 1-1-1 Hondo, Akita 010-8543, Japan; 4 Department of Pharmacokinetics, Akita University Graduate School of Medicine, 1-1-1 Hondo, Akita 010-8543, Japan

**Keywords:** everolimus, polymorphism, pregnane X-receptor, P-glycoprotein

## Abstract

The purpose of this study was to evaluate the effects of *NR1I2* (7635G>A and 8055C>T) and *ABCB1* (1236C>T, 2677G>T/A, and 3435C>T) genetic polymorphisms on everolimus pharmacokinetics in 98 Japanese renal transplant patients. On day 15 after everolimus administration, blood samples were collected just prior to and 1, 2, 3, 4, 6, 9, and 12 h after administration. The dose-adjusted area under the blood concentration–time curve (AUC_0-12_) of everolimus was significantly lower in patients with the *NR1I2* 8055C/C genotype than in those with other genotypes (*p* = 0.022) and was significantly higher in male patients than female patients (*p* = 0.045). Significant correlations between the dose-adjusted AUC_0-12_ of everolimus and age (*p* = 0.001), aspartate transaminase (*p* = 0.001), and alanine transaminase (*p* = 0.005) were found. In multivariate analysis, aging (*p* = 0.008) and higher alanine transaminase levels (*p* = 0.032) were independently predictive of a higher dose-adjusted everolimus AUC_0-12_. Aging and hepatic dysfunction in patients may need to be considered when evaluating dose reductions in everolimus. In renal transplant patients, management using everolimus blood concentrations after administration may be more important than analysis of *NR1I2* 8055C>T polymorphism before administration.

## 1. Introduction

Everolimus, a mammalian target of rapamycin inhibitor, has been approved for the prophylaxis of acute rejection in renal transplant recipients [1,2,3,4,5]. Individual variability in blood concentrations of everolimus involves several factors, including genetic factors and drug interactions [6]. Everolimus is metabolized by cytochrome P450 (CYP) 3A4 and CYP3A5 in the gut and liver, although CYP3A4 contributes more to this process than CYP3A5 [7]. In several studies, the pharmacokinetics of everolimus have been reported to independent of *CYP3A5* polymorphism [6,7,8,9,10].

Everolimus is also a substrate of P-glycoprotein (encoded by *ABCB1*) in the intestines. The calcineurin inhibitor cyclosporine, an inhibitor of P-glycoprotein, significantly increases the area under the blood concentration–time curve (AUC) and the maximum blood concentration (C_max_) of everolimus but does not affect the elimination half-life [11], and these pharmacokinetic parameters support that drug interactions between cyclosporine and everolimus occur in the intestines [11]. Thus, P-glycoprotein inhibitors primarily affect the oral bioavailability of everolimus rather than everolimus clearance, highlighting the key role of intestinal P-glycoprotein. The three most common single nucleotide polymorphisms (SNPs) identified in the *ABCB1* transporter are 1236C>T, 2677G>T/A, and 3435C>T [12]; however, in a previous study using a population pharmacokinetic model of 53 renal transplant recipients, *ABCB1* 1236C>T, 2677G>T/A, and 3435C>T polymorphisms did not affect the apparent oral clearance of everolimus [9]. P-glycoprotein and CYP3A is regulated by activated pregnane X-receptor (PXR, *NR1I2*), an important nuclear receptor [13,14], and the 7635G>A (rs6785049) and 8055C>T (rs2276706) polymorphisms in the *NR1I2* gene for human PXR are associated with altered CYP3A4 regulation [15]. However, in this previous study using a pharmacokinetic model [9], *NR1I2* 7635G>A and 8055C>T also did not affect the apparent oral clearance of everolimus. Until now, the effects of *NR1I2* and *ABCB1* genetic polymorphisms on pharmacokinetic parameters such as the AUC and C_max_ to evaluate the involvement of intestinal P-glycoprotein have been unclear. Therefore, the clinical study using pharmacokinetic parameters for the absorption process, but not the apparent clearance, of everolimus is necessary.

Although everolimus can be utilized after renal transplantation to reduce nephrotoxicity induced by tacrolimus [1,2,3], the blood concentrations of everolimus are not influenced by tacrolimus but are affected by cyclosporine [10,16,17,18]. Therefore, the dose of everolimus that achieves equivalent exposure is 1.5- or 2-fold higher in the presence of tacrolimus than in the presence of cyclosporine [11,17,18]. Accordingly, when everolimus is administered in combination with cyclosporine, the influence of genetic polymorphisms, such as polymorphisms in *NR1I2* or *ABCB1*, on everolimus pharmacokinetics cannot be sufficiently assessed. In a previous study using a population pharmacokinetic model [9], the blood concentrations of everolimus after beginning treatment at an initial dose of 3 mg twice daily were adjusted based on the target trough blood concentration (C_0_) of 6–8 ng/mL when a calcineurin inhibitor-free monotherapy regimen was switched from an immunosuppressive regimen including cyclosporine [9]. Moreover, when everolimus was used in combination with other immunosuppressive drugs, such as calcineurin inhibitors and glucocorticoids, the C_0_ range was generally set to 3–8 ng/mL [6]. Until now, in renal transplant recipients, the effects of *NR1I2* and *ABCB1* genetic polymorphisms on pharmacokinetics of everolimus in combination with tacrolimus have remained unclear. In addition, we cannot evaluate the influence of P-glycoprotein on an absorption process of everolimus using only one point of the C_0_. Many studies have investigated the influence of *ABC* transporter polymorphisms by using only one point of the everolimus C_0_ [19,20,21]. 

Accordingly, in this study, we evaluated the effects of *NR1I2* and *ABCB1* genetic polymorphisms on everolimus pharmacokinetics in 98 Japanese renal transplant patients in combination with tacrolimus. 

## 2. Results

The clinical characteristics of the patients prior to initiation of everolimus therapy are listed in Table 1. The median age was 54.5 years, and the median body weight was 58.7 kg. The genotype frequencies for the *NR1I2* (7635G>A and 8055C>T) and *ABCB1* (1236C>T, 2677G>T/A, and 3435C>T) genetic polymorphisms in 98 Japanese renal transplant patients are shown in Table 1. 

**Figure 1:**
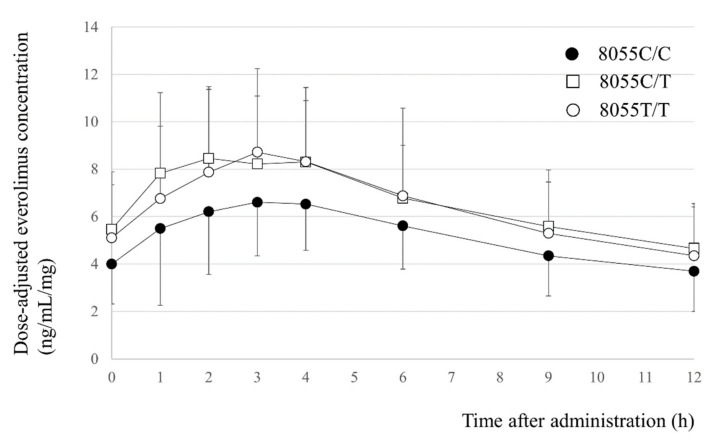
Mean (± standard deviation) plasma concentration–time profiles of everolimus in renal transplant recipients with the NR1I2 8055C/C (closed circles, *n* = 20), 8055C/T (open squares, *n* = 52), or 8055T/T genotype (open circles, *n* = 26) on day 15 after initiation of everolimus treatment at a dose of 0.75 mg twice daily (1.5 mg daily dose) in combination with tacrolimus.

On day 15 at a steady-state after beginning treatment at an initial everolimus dose of 0.75 mg twice daily (1.5 daily dose), the everolimus C_0_ was significantly correlated with the AUC_0-12_ (slope = 10.978, intercept = 15.710, *r^2^* = 0.862, *p* < 0.001). The dose-adjusted C_0_ and AUC_0-12_ of everolimus in patients with the *NR1I2* 8055C/C genotype were significantly lower than those in patients with the 8055C/T or 8055T/T genotype (*p* = 0.011 and 0.022, respectively); however, there were no significant differences in the elimination half-life among the three groups (Figure 1 and Table 2). The geometric mean dose-adjusted C_0_ and AUC_0-12_ of everolimus in patients with the *NR1I2* 8055C/C, 8055C/T and 8055T/T genotype were 3.7, 5.2, and 4.4 ng/mL/mg, respectively (*p* = 0.011, one-way ANOVA test), and 60.0, 76.9, and 71.7 ng·h/mL/mg, respectively (*p* = 0.027, one-way ANOVA test). In addition, there were significant differences in the dose-adjusted trough blood concentrations at 12 h after everolimus administration (C_12_) and the elimination half-life of everolimus among the three genotype groups of *ABCB1* 2677G>T/A (*p* = 0.042 and 0.035, respectively); however, blood concentrations of everolimus in heterozygous carriers of the *ABCB1* 2677 T or A allele were the highest (Table 2).

The dose-adjusted C_0_ and AUC_0-12_ values of everolimus in male patients were significantly higher than those in female patients (*p* = 0.004 and 0.045, respectively; Table 3). Significant correlations were observed between the dose-adjusted C_0_ of everolimus on day 15 after beginning therapy and age (*p* < 0.001), body weight (*p* = 0.028), aspartate transaminase (*p* < 0.001), alanine transaminase (*p* < 0.001), and total bilirubin (*p* = 0.039; Table 3). Furthermore, significant correlations were found between the dose-adjusted AUC_0-12_ of everolimus and age (*p* = 0.001), aspartate transaminase (*p* = 0.001), and alanine transaminase (*p* = 0.005; Table 3).

The results of multiple regression analyses, including covariate analyses, are listed in Table 4. Aging (*p* = 0.001), higher alanine transaminase value (*p* = 0.019), and body weight (*p* = 0.027) were independently predictive of a higher dose-adjusted everolimus C_0_. In addition, aging (*p* = 0.008) and higher alanine transaminase value (*p* = 0.032) were independently predictive of a higher dose-adjusted everolimus AUC_0-12_. However, the determination coefficients for the everolimus C_0_ and AUC_0-12_ were low (0.164 and 0.100, respectively; Table 4).

There were significant differences in sex; everolimus C_0_ and AUC_0-12_ on day 15; single dose of everolimus at 1 year; and patient age between patients with dose reduction in everolimus within 1 year based on the target C_0_ range and patients with no change in dose (Table 5). However, there were no significant differences between genotypes of *NR1I2* and *ABCB1* (Table 5). On the other hand, there were no significant differences in the everolimus C_0_ or AUC_0-12_ on day 15 between patients with dose reduction in everolimus within 1 year based on the onset of everolimus-induced side effects, such as stomatitis and leukopenia and patients with no change in dose (Table 5). Although everolimus was used in combination with tacrolimus, the changes in everolimus dose within 1 year after beginning everolimus therapy were caused by being above the target concentration range of tacrolimus. There were no significant differences in tacrolimus C_0_ and AUC_0-24_ between each group. The dose reductions in everolimus by the onset of side effects or above the target C_0_ range could not be predicted from information of *NR1I2* and *ABCB1* genetic polymorphisms.

## 3. Discussion

To the best of our knowledge, this is the first study to report the effects of *NR1I2* and *ABCB1* genetic polymorphisms on the actual AUC_0-12_ of everolimus calculated using many sampling points with larger numbers of patients. In the clinical concertation range of everolimus, a univariate analysis of 98 renal transplant recipients showed that the dose-adjusted C_0_ and AUC_0-12_ values of everolimus in patients with the *NR1I2* 8055C/C genotype were significantly lower than those in patients with the 8055C/T or 8055T/T genotype; however, in the multivariate analysis, *NR1I2* and *ABCB1* polymorphisms did not affect interindividual variability in everolimus blood concentrations. In multivariate analyses, age and alanine transaminase values had major effects on everolimus C_0_ and AUC_0-12_. In addition, the age of patients who underwent dose reductions in everolimus within 1 year was significantly higher than that in patients who did not undergo dose changes. Therefore, everolimus dose reductions should be considered as patients age and in patients with hepatic dysfunction.

In patients with mild and moderate hepatic impairment, the dose of everolimus should initially be reduced [22]. After the initial dose reduction, the dose of everolimus may be adjusted based on the target blood concentration of everolimus [6,22]. Therefore, for renal transplant recipients, based on alanine transaminase values of recipients before everolimus administration, the clinician adjust the initial dose of everolimus (0.25 or 0.5 mg twice daily) and reduce it from the standard dose (0.75 mg twice daily). In the univariate analysis in the current study, the dose-adjusted C_0_ and AUC_0-12_ values of everolimus in male patients were significantly higher than those in female patients; however, in the multivariate analysis, sex difference did not affect variability in everolimus blood concentrations. In our study, the alanine transaminase values of male patients were significantly higher than that of female patients (*p* < 0.001). Thus, patient backgrounds seem to be a cause of sex difference, and in the multivariate analysis, sex difference was excluded. Consequently, careful monitoring of alanine transaminase values for renal transplant recipients, especially for elderly patients, is necessary.

In a previous study, patient age and body weight did not contribute to interindividual variability in everolimus blood concentrations [23], in contrast to the results of our current study. Additionally, in this previous study, the mean patient age was 44.4 years [23], which was much younger than that (52.8 years, median 54.5 years) in the current study. Moreover, the mean body weight of patients in the previous study was higher than that in the current study (76.7 versus 60.7 kg, respectively) [23]. Thus, the Japanese patients included in our study were older and had lower body weights, and these factors may have resulted in differences in everolimus exposure. Similar to our study, another previous study in Japanese renal transplant recipients (median age, 51 years) showed that the dose-adjusted everolimus C_0_ was affected by patient age [24]. Furthermore, the expression of P-glycoprotein in intestinal tissue was not correlated with patient age (in patients 21–67 years old) [25]. Therefore, reductions in the drug-metabolizing capacity of the liver observed during aging may increase everolimus exposure.

Four previous clinical studies demonstrated the effects of *ABCB1* genetic polymorphisms on everolimus pharmacokinetics in 53 renal [9], 24 renal [19], 37 cardiac [20], and 65 lung transplant recipients [21]. Although only everolimus C_0_ was used in these studies [19,20,21], in all of these studies, *ABCB1* genetic polymorphisms did not affect everolimus blood concentrations. Therefore, *ABCB1* genotyping prior to the initiation of everolimus therapy is not recommended [6], consistent with the findings of our current study. Because the activation of PXR induces the expression of drug-metabolizing enzymes, such as CYP3A and ABC transporters (e.g., P-glycoprotein) [13,14,26], differences in PXR activation may influence interindividual variability in everolimus pharmacokinetics because everolimus is a substrate of both CYP3A and P-glycoprotein. In the univariate analysis, the dose-adjusted C_0_ and AUC_0-12_ values of everolimus in patients with the *NR1I2* 8055C/C genotype were significantly lower than those in patients with other genotypes; however, no changes in the elimination half-life were observed among genotypes. This phenomenon suggests that intestinal P-glycoprotein affects everolimus absorption. However, in the multivariate analysis, *NR1I2* polymorphisms did not affect the blood concentrations of everolimus. By contrast, blood concentrations of everolimus seemed to be more strongly influenced by aging and liver function than by the *NR1I2* 8055C>T polymorphism. Consequently, our current results using actual pharmacokinetic parameters of everolimus obtained at eight time points were consistent with the results obtained from a previous study using a population model [9]. Thus, the pharmacokinetics of everolimus in renal transplant recipients cannot be predicted based on drug metabolism and transport-related SNPs. Management using everolimus blood concentrations after administration may be more important than an analysis of drug metabolism and transport-related SNPs before everolimus administration. Similar to the previous reports [9,27], the development of a population pharmacokinetic mode will be necessary to improve the precision of the therapeutic drug monitoring of everolimus. Further study for population pharmacokinetic mode development using our everolimus pharmacokinetic data will be necessary.

## 4. Materials and Methods

### 4.1. Patients and Protocols

Ninety-eight Japanese renal transplant recipients (34 women and 64 men) who received renal grafts at Akita University Hospital between October 2013 and June 2021 were enrolled in the retrospective study. The study protocol was approved by the Ethics Committee of Akita University Graduate School of Medicine (approval no. 1140), and all patients provided written informed consent. The study was carried out during hospitalization.

The criteria for eligibility for the study were as follows: (1) patients were treated with an immunosuppressive regimen based on tacrolimus (Graceptor; Astellas, Tokyo, Japan), mycophenolate mofetil (MMF; Cellcept; Chugai Pharmaceutical, Tokyo, Japan), and steroids, and on day 15 after renal transplantation, everolimus (Certican; Novartis Pharma, Tokyo, Japan) was added; (2) patients received tacrolimus every 24 h at the designated time (09:00 AM), and MMF and everolimus in equally divided doses every 12 h at designated times (09:00 AM and 21:00 PM); (3) patients without serious hepatic dysfunction, renal dysfunction, or gastrointestinal motility; (4) patients who were not taking concomitant drugs, supplements, or foods that may affect CYP3A or P-glycoprotein function; (5) nonsmokers; and (6) patients with an ABO compatible blood type.

All patients received everolimus 0.75 mg twice daily (1.5 mg daily dose) as the initial dose. The target C_0_ of everolimus was 3–5 ng/mL after the second week [17]. The target C_0_ values of tacrolimus were 10–12 ng/mL during the first week, 8–10 ng/mL during the second to fourth weeks after renal transplantation, and 5–8 ng/mL thereafter. Methylprednisolone was given concomitantly at a dose of 500 mg intravenously (i.v.) on the day of surgery and was tapered to 40 mg/day i.v. during the first week. Subsequently, 10–15 mg/day oral prednisolone was administered in the second to third weeks and 7.5–10 mg/day oral prednisolone was administered thereafter.

Everolimus dose reductions within 1 year were carried out based on the grades of reported side effects, such as stomatitis and leukopenia, and on C_0_ values of above the target range of 5.0 ng/mL. By contrast, increased everolimus doses were administered based on the target everolimus C_0_ of 3.0 ng/mL.

### 4.2. Sample Collection and Analytical Methods

On day 15 after everolimus administration (namely, day 29 after renal transplantation), whole blood samples were collected by venipuncture just prior to (C_0_) and at 1, 2, 3, 4, 6, 9, and 12 h (C_12_) after everolimus and tacrolimus administration at 09:00 AM. In addition, for tacrolimus, whole blood samples were also collected at 24 h after administration. Thereafter, blood concentrations of everolimus and tacrolimus were determined by electrochemiluminescence immunoassay using a Cobas e411 system (Roche, Tokyo, Japan) and chemiluminescence magnetic microparticle immunoassays on an Architect-i1000 system (Abbott Laboratories, Abbott Park, IL, USA), respectively, according to the manufacturers’ instructions.

### 4.3. Genotyping

DNA was extracted from peripheral blood samples with a QIAamp Blood Kit (Qiagen, Hilden, Germany) and stored at −80 °C until analysis. Genotyping procedures identifying the C and T alleles in exon 12 (1236C>T, rs1128503), the G and T/A alleles in exon 21 (2677G>T/A, rs2032582), and the C and T alleles in exon 26 (3435C>T, rs1045642) of the *ABCB1* gene [28,29,30]; the G and A alleles in intron 5 (7635G>A, rs6785049) and the C and T alleles in intron 6 (8055C>T, rs2276707) of the *NR1I2* gene [31,32] were identified using polymerase chain reaction–restriction fragment length polymorphism.

### 4.4. Pharmacokinetic Analysis

Pharmacokinetic analyses of everolimus were carried out using a standard noncompartmental method with Phoenix WinNonlin 6.4 (Pharsight Co., Mountain View, CA, USA). The AUC_0–12_ was calculated using the linear trapezoidal rule. The C_max_ and C_0_ were obtained directly from the profile. The elimination half-life was obtained using the log-linear regression of the terminal phase of the concentration-time data with at least 3 sampling points (elimination half-life = ln2/ke; where ke = elimination rate constant).

### 4.5. Statistical Procedures

Kolmogorov–Smirnov tests were used to assess distributions. The clinical characteristics of renal transplant recipients were expressed as medians (quartile 1–quartile 3) or numbers. Kruskal–Wallis tests or Mann–Whitney U tests were used to elucidate differences between groups. Spearman’s rank correlation coefficient test was used to assess correlations in continuous values between groups, and all results were expressed as correlation coefficients (*r* values). The effects of factors in univariate analysis were evaluated using stepwise multiple linear regression analysis. Variables with borderline significance (*p* < 0.2) on the univariate analysis were subjected to multivariate regression analyses. Dummy variables were used to replace the groups (1 and 0 in 2 groups; 1 and 0, 0 and 0, and 0 and 1 in 3 groups). Results with *p* values of less than 0.05 were considered significant, and SPSS 20.0 for Windows (SPSS IBM Japan Inc., Tokyo, Japan) was used for all statistical analyses.

## 5. Conclusions

Age and alanine transaminase values had major effects on everolimus C_0_ and AUC_0-12_. Therefore, aging and hepatic dysfunction should be considered when evaluating the need for everolimus dose reduction. Management using blood concentrations of everolimus after administration may be more important than analysis of drug metabolism and transport-related genetic polymorphisms before everolimus administration. Especially for elderly renal transplant recipients, careful monitoring of everolimus blood concentrations and alanine transaminase values is necessary.

## Figures and Tables

**Table 1:** Clinical characteristics of patients before beginning everolimus therapy.

Numbers of Patients (Female: Male)	98	(34: 64)
Age (years)	54.5	(44.0–63.3)
Body weight (kg)	58.7	(50.4–66.5)
Laboratory test values		
Aspartate transaminase (IU/L)	14.0	(11.0–16.0)
Alanine transaminase (IU/L)	14.0	(10.0–20.0)
Serum albumin (g/dL)	3.8	(3.5–4.0)
Total bilirubin (mg/dL)	0.4	(0.4–0.6)
Serum creatinine (mg/dL)	1.3	(1.0–1.7)
*NR1I2* 7635G>A (rs6785049) G/G: G/A: A/A	32: 48: 18	
*NR1I2* 8055C>T (rs2276707) C/C: C/T: T/T	20: 52: 26	
*ABCB1* 1236C>T (rs1128503) C/C: C/T: T/T	10: 44: 44	
*ABCB1* 2677G>T/A (rs2032582) G/G: G/T+G/A: T/T+T/A	27: 53: 18	
*ABCB1* 3435C>T (rs1045642) C/C: C/T: T/T	36: 42: 20	

Data are presented as the median (quartile 1–quartile 3) or number.

**Table 2:** Pharmacokinetic parameters of everolimus in *NR1I2* and *ABCB1* genotype groups.

***NR1I2* 7635G>A (rs6785049)**	**G/G**		**G/A**		**A/A**		***p*–value**
Numbers of patients	32		48		18		
C_0_/D (ng/mL/mg)	4.6	(3.4–6.8)	5.0	(3.9–6.2)	3.6	(2.9–5.6)	0.245
C_12_/D (ng/mL/mg)	4.0	(2.9–6.2)	4.2	(3.4–5.6)	3.4	(2.4–5.3)	0.385
C_max_/D (ng/mL/mg)	10.1	(6.9–11.9)	9.0	(7.9–11.6)	8.6	(6.1–11.4)	0.716
Half–life (h)	7.9	(5.8–9.0)	7.7	(6.1–10.0)	7.0	(6.0–10.6)	0.119
AUC_0-12_/D (ng·h/mL/mg)	76.3	(54.9–100)	75.5	(63.6–89.9)	63.2	(48.5–80.9)	0.371
***NR1I2* 8055C>T (rs2276707)**	**C/C**		**C/T**		**T/T**		***p*–value**
Numbers of patients	20		52		26		
C_0_/D (ng/mL/mg)	3.4	(2.7–5.1)	5.3	(3.9–6.7)	4.5	(3.1–7.0)	0.011
C_12_/D (ng/mL/mg)	3.2	(2.2–5.3)	4.4	(3.5–5.9)	3.9	(3.0–6.1)	0.069
C_max_/D (ng/mL/mg)	7.6	(6.0–9.7)	9.9	(7.9–12.0)	10.0	(8.0–11.4)	0.057
Half–life (h)	7.0	(5.4–9.8)	8.0	(6.4–10.5)	7.0	(5.7–8.3)	0.119
AUC_0-12_/D (ng·h/mL/mg)	62.0	(43.5–75.0)	82.7	(64.1–97.9)	66.4	(59.7–90.8)	0.022
***ABCB1* 1236C>T (rs1128503)**	**C/C**		**C/T**		**T/T**		***p*–value**
Numbers of patients	10		44		44		
C_0_/D (ng/mL/mg)	5.0	(4.2–6.9)	4.3	(3.2–6.4)	5.0	(3.4–6.5)	0.646
C_12_/D (ng/mL/mg)	4.2	(3.9–5.9)	3.6	(2.6–5.3)	4.3	(3.1–6.0)	0.321
C_max_/D (ng/mL/mg)	9.6	(8.3–12.7)	8.5	(6.7–9.5)	9.8	(7.8–11.4)	0.341
Half–life (h)	6.9	(5.7–9.8)	7.2	(6.0–9.5)	7.8	(6.3–9.8)	0.526
AUC_0-12_/D (ng·h/mL/mg)	82.4	(67.7–89.1)	69.3	(54.1–87.6)	74.6	(57.3–98.2)	0.321
***ABCB1* 2677G>T/A (rs2032582)**	**G/G**		**G/T+G/A**		**T/T+T/A**		***p*–value**
Numbers of patients	27		53		18		
C_0_/D (ng/mL/mg)	4.4	(2.8–5.3)	5.1	(3.7–7.0)	4.4	(3.7–6.3)	0.109
C_12_/D (ng/mL/mg)	3.5	(2.6–4.6)	4.4	(3.3–6.2)	3.8	(2.7–5.6)	0.042
C_max_/D (ng/mL/mg)	8.9	(6.6–11.1)	9.7	(7.8–12.0)	8.8	(7.2–10.5)	0.583
Half–life (h)	6.4	(5.4–8.4)	8.0	(6.5–10.0)	7.5	(6.1–9.7)	0.035
AUC_0-12_/D (ng·h/mL/mg)	64.0	(52.9–84.0)	76.4	(61.9–99.3)	67.7	(54.4–87.4)	0.110
***ABCB1* 3435C>T (rs1045642)**	**C/C**		**C/T**		**T/T**		***p*–value**
Numbers of patients	36		42		20		
C_0_/D (ng/mL/mg)	4.8	(3.7–6.7)	4.2	(3.3–6.5)	5.0	(3.9–6.2)	0.530
C_12_/D (ng/mL/mg)	4.0	(3.2–5.6)	3.8	(2.7–6.7)	4.6	(3.2–5.9)	0.660
C_max_/D (ng/mL/mg)	10.0	(6.9–12.1)	9.2	(7.0–11.2)	8.8	(7.9–12.1)	0.654
Half–life (h)	6.9	(5.5–10.0)	7.7	(6.2–9.6)	7.8	(6.5–9.3)	0.728
AUC_0-12_/D (ng·h/mL/mg)	77.8	(60.0–88.0)	68.1	(55.9–94.3)	74.6	(63.6–96.4)	0.659

Data are presented as the median (quartile 1–quartile 3) or number. C_0_, trough blood concentration at morning time; C_12_, trough blood concentration at nighttime of 12 h after administration; C_max_, maximum blood concentration; AUC_0-12_, area under the blood concentration–time curve from 0 to 12 h; D, single dose. Kruskal–Wallis test.

**Table 3:** Comparison of everolimus dose-adjusted C_0_ and AUC_0-12_ values and clinical characteristics of patients.

**Clinical Characteristics**	***n***	**Dose-Adjusted C_0_ (ng/mL/mg)**		***p* Value**
		**Median**	**Quartile 1–3**	
Sex				
Female	34	3.7	(2.7–5.5)	0.004 ^a^
Male	64	5.1	(4.2–6.6)	
	Correlation coefficient (*r*)			
Age (years)	0.359			<0.001
Body weight (kg)	0.223			0.028
Laboratory test values				
Aspartate transaminase	0.364			<0.001
Alanine transaminase	0.356			<0.001
Serum albumin	−0.034			0.740
Total bilirubin	0.209			0.039
Serum creatinine	0.055			0.592
**Clinical Characteristics**	***n***	**Dose-Adjusted AUC_0-12_ (ng·h/mL/mg)**		***p* Value**
		**Median**	**Quartile 1–3**	
Sex				
Female	34	63.3	(51.1–87.0)	0.045 ^a^
Male	64	77.2	(63.2–96.0)	
	Correlation coefficient (*r*)			
Age (years)	0.327			0.001
Body weight (kg)	0.159			0.118
Laboratory test values				
Aspartate transaminase	0.328			0.001
Alanine transaminase	0.283			0.005
Serum albumin	−0.067			0.512
Total bilirubin	0.170			0.094
Serum creatinine	0.041			0.686

^a^ Mann–Whitney test.

**Table 4:** Stepwise multiple regression analysis of explanatory variables for everolimus dose-adjusted C_0_ and AUC_0-12_ values.

**Explanatory Variable for Everolimus C_0_**	**Slope**	**SE**	**SRC**	***p* Value**	***R* ^2^**
Age (years)	0.055	0.016	0.318	0.001	0.164
Alanine transaminase (IU/L)	0.047	0.020	0.224	0.019	
Body weight (kg)	0.031	0.014	0.216	0.027	
Intercept =	−0.500	1.336			
**Explanatory Variable for Everolimus AUC_0-12_**	**Slope**	**SE**	**SRC**	***p* value**	***R* ^2^**
Age (years)	0.548	0.202	0.262	0.008	0.100
Alanine transaminase (IU/L)	0.537	0.246	0.210	0.032	
Intercept =	38.115	11.518			

SE, standard error; SRC, standardized regression coefficient.

**Table 5:** Relationships between changes in everolimus dose within 1 year after beginning everolimus therapy and genotypes of *NR1I2* and *ABCB1*.

Change of Everolimus Dose Within 1 Year	Onset of Side Effects		Dose Adjusted Based on Target Range of Everolimus C_0_					
	Dose Reduction or Withdrawal		Dose Reduction		No Change		Increase in Dose	
Numbers of patients (Female: male)	27	(9: 18)	22	(3: 19) *	37	(16: 21)	12	(6: 6)
C_0_ on day 15 (ng/mL)	3.9	(3.1–5.4)	4.3	(3.6–6.2) **	3.4	(2.7–3.9)	2.7	(2.1–3.2)
AUC_0-12_ on day 15 (ng·h/mL)	59.1	(48.4–74.4)	70.8	(53.1–78.6) **	51.8	(45.8–63.3)	40.9	(34.2–46.8) *
Starting single dose (mg, baseline)	0.75		0.75		0.75		0.75	
Single dose at 1 year (mg)	0.5	(0–0.5) ***	0.5	(0.25–0.5) ***	0.75		1.0	(1.0–1.2) ***
Tacrolimus C_0_	7.6	(6.7–9.1)	7.8	(5.6–8.5)	7.0	(5.7–8.8)	8.2	(6.7–9.0)
Tacrolimus AUC_0-24_	266	(220–297)	273	(225–315)	269	(215–320)	271	(245–338)
Age (years)	54.0	(44.0–63.0)	58.5	(55.5–65.0) *	51.0	(39.5–63.0)	49.5	(40.0–60.0)
Body weight (kg)	59.7	(46.6–76.5)	59.9	(55.4–65.7)	54.5	(46.9–64.0)	57.9	(51.7–64.6)
Alanine transaminase (IU/L)	15.0	(12.0–22.0)	15.0	(10.0–25.3)	14.0	(8.5–20.0)	11.5	(9.3–13.0)
*NR1I2* 7635G>A, G/G: G/A: A/A	9: 14: 4		5: 11: 6		11: 21: 5		7: 2: 3	
*NR1I2* 8055C>T, C/C: C/T: T/T	3: 17: 7		8: 10: 4		6: 21: 10		3: 4: 5	
*ABCB1* 1236C>T, C/C: C/T: T/T	3: 8: 16		2: 11: 9		4: 19: 14		1: 6: 5	
*ABCB1* 2677G>T/A, G/G: G/T+G/A: T/T+T/A	6: 14: 7		4: 14: 4		14: 18: 5		3: 7: 2	
*ABCB1* 3435C>T, C/C: C/T: T/T	9: 11: 7		8: 9: 5		13: 17: 7		6: 5: 1	

Data are presented as the median (quartile 1–quartile 3) or number. Target range of everolimus C_0_: 3–5 ng/mL. * *p* < 0.05, ** *p* < 0.01, *** *p* < 0.001 compared with the no change group.

## Data Availability

Data and materials are available on request from the corresponding author.
